# Icteroid Fever

**Published:** 1885-11-20

**Authors:** B. Frank Humphreys

**Affiliations:** Texas


### ICTEROID FEVER.

BY B. FRANK HUMPHREYS, M.D., TEXAS.

On the morning of the 26th of September, I was seized with a chill, which was followed by fever, which seemed more prostrating than an ordinary chill and fever. In the evening I took two improved cathartic pills which acted well, and, in the after part of the night, I took 10 to 20 grains of quinine.

Next morning, feeling all right, I responded to a call to visit a sick child, only a short distance from my residence. After this I went to the hotel to visit other patients and soon returned home feeling unusually bad. I partook of a light breakfast and soon afterwards took my bed, when, to my surprise, I had another chill, even harder than the preceding one. I suffered a great deal, and was unable to leave my bed during the day.

---

In the evening, I took the following preparation :

℞. Calomel..........................................iv grains,
Euonymin, leptandria, each..................iv grains,
Rhebarb..........................................xv grains.

Mix, and divide into three capsules.

One every two hours.

At midnight I took about 10 grains of quinine ; the same at 3 and 6 a. m—making about 30 grains. The calomel acted twice only during the night.

Soon after taking the last dose of quinine my feelings were indescribable, when another chill, harder than either of the preceding ones, followed to my surprise and confusion. Shortly after the chill I felt inclined to micturate, when, lo ! the excretion was that dark, wine-colored urine that is so characteristic or pathonomic of this dreadful disease, of which I was at last a victim myself ! I cannot express the terror and despondency such a sight produced upon me, and how doubtful I felt of my recovery, considering my advanced age of fifty-four years.

Nevertheless, I took courage and determined to try to obey, for a while, at least, the injunction : "Physician, heal Thyself !"

Being unable to dose out medicine, I had my daughter to prepare a saturated solution of chlorate of potassium in a tumbler. Of this I took a tablespoonful, which was followed, in an hour, with one teaspoonful of sweet spirits of nitre, well diluted with water. An hour after the nitre, I took a good swallow of Cognac brandy, in which was suspended willow charcoal (Ellis'), the brandy slightly diluted and sweetened. Each dose containing about a teaspoonful of charcoal.

These remedies were alternated, as stated, every hour during the day (Saturday), as the fever was very high all that day and night, the temperature reaching 105° Fah. A faithful nurse bathed my face, head and forearms in ice-water, and favored evaporation by fanning, which was kept up all day and night. The urinary discharge was often and very free, without pain. By Sunday morning the color of the urine was not so dark nor quite so free, nor did it appear to contain red blood corpuscles, as at first, but looked more like dark, bilious matter passing through the kidneys, which some physicians claim is simply bilurea (bilirubine and biliverdine).

Fearing that I might soon get in a condition to be unable to direct treatment, I had all my neighboring physicians summoned to my bedside Saturday morning, but, all being absent, I was not visited by a physician until evening, when Drs. O. A. Fitts and son responded, and continued their visits several times a day until Tuesday, when they discontinued their visits. The elder Dr. Fitts, being a very successful practitioner in this disease, saw at once that the treatment I had outlined was good, and advised its continuance—which was done, the interval between doses being lengthened as the symptoms improved.

On Sunday morning, September 29, the skin had assumed that bronzed appearance which always accompanies this fever, while the whites of the eyes presented that yellow color always observed in this disease and jaundice ; but neither the skin nor the eyes were as deeply colored as usual. The urinary discharge continued quite freely and was still very dark, wherefore the attending physician advised one teaspoonful fluid extract of ergot to be repeated as often as necessary. It was taken, but its sickening odor, and the irritable condition of my stomach, caused its immediate ejection, wherefore I did not attempt to take any more. By this time the stomach was so sensitive to all medicines it was almost impossible to retain anything, on account of which the remedies mentioned in the preceding part of this paper were taken at long intervals ; and by Monday, the 30th, I could not tolerate anything like medicine—my condition having improved so much, little was needed.

Sunday night a number of dark bilious actions from the bowels, apparently the effects of the medicine taken Friday evening, seemed to give much relief, though a tenderness, and feeling of soreness in the stomach and bowels supervened, the discharges continuing until Monday evening. Up to this time I had not taken a mouthful of nourishment, except milk and brandy mixed—which I had to use quite freely at times, being threatened with collapse, as indicated by the cold, heavy perspiration, small, feeble pulse, etc., all of which were carefully watched by a kind friend and neighbor, who administered the stimulant as seemed necessary, and, doubtless, thereby preserved me from dissolution.

During all this time I had not enjoyed a good, sound sleep ; in consequence of which, and the prostrating tendency of the fever, I was unable to speak above a whisper, and conversation directed to me, or within my hearing, was very annoying.

Tuesday evening, September 31, I was removed to a more pleasant room, unnanoyed by company. On account of the lax and tender condition of the bowels, I took a sound dose of opium and drank freely of my neighbor's excellent home-made blackberry wine, wherefore I had a most pleasant night of refreshing sleep, and awoke next morning feeling greatly renovated, with a blessing for the ruby wine, and for opium, the soothing balm of weary nature. I did not hesitate to take the opium, nor fear any evil effects upon the kidneys, as it was urgently demanded and accomplished great good, as the urinary secretion continued as before, without any decrease.

From this time on I improved daily, and was able to take liquid nourishment, my appetite increasing all the while, though my strength, even now, after two weeks of convalescence, has not improved much, notwithstanding I am taking the tincture of the chloride of iron, and two parvules of strychnia, thrice daily, an hour after each meal, and brandy when feeling exceedingly weak.

A week after beginning to convalesce my urine was so much increased in quantity—necessitating my rising severul times every night to empty the bladder—I determined to test it for sugar and albumen, as I suspected something of the kind. I was delighted to find that there was neither sugar nor albumen, but, on the contrary, it gave every indication of being normal.

Having written a somewhat exhaustive article on this subject, which appeared in the Nashville Journal of Medicine and Surgery, March, 1874; and as the treatment outlined in that article differs so materially from that given in this paper, it may be necessary to add a word of explanation.

The treatment which seemed so successful a decade ago is not, generally, used now, though there are many physicians who still hold to the quinine treatment, but it is evidently not so satisfactory as the plan herein described. Patients recover under each method of treatment, and they likewise die under each plan of treatment, but many more recover without quinine than with it, and there need be no apprehension of another chill or rigor after the disease is once fully ushered in, even if the quinine is discontinued. I have never known a chill to follow the suspension of the quinine, provided the treatment already described herein is faithfully applied. I was an earnest advocate for the quinine treatment ten years ago, and it appeared to be the sheet-anchor as the disease then manifested itself with frequent rigors, which seemed to yield to quinine and brandy.

I may have been laboring under an error then, as many are now who use the quinine treatment; but, if wrong, I take this opportunity to right myself, feeling sure that "an honest confession is good for the soul."

My attention was first directed to the evil effects of quinine in this disease by noticing its effects upon a favorite child, who had several attacks annually for four or five years, beginning when she was only five years old. Very soon after taking an antiperiodic dose of quinine she would be seized with violent rigors, followed by hematuria, bronzed skin and the other characteristic symptoms of the disease. Upon suspension of the quinine, her condition invariably improved by using the treatment already detailed in this article. It made no difference about preparing the system for quinine by hepatic remedies, the results stated always followed its use. The same child is subject to frequent attacks of fever now, but I do not dare to give her a grain of quinine. I do not argue that quinine produces the disease; it might arise without it; but, when once ushered in, the patients do much better without the quinine.

I still hold to the pathological views expressed in the previous article alluded to, nor have I observed any facts to change my mind in reference to its clinical history, ætiology, symptoms, diagnosis, pathology, prognosis, and prophylaxis. The only change of opinion is with regard to the treatment, and I feel very confident the plan herein outlined is worthy of acceptance until something better is tested and proven to be more successful.

It is a hard struggle for an old physician to give up a favorite method of practice; and not until forced to do so, by stubborn facts, will he abandon his hobbies. An old retired physician, whose son is just recovering from icteroid fever, informs me that he was wedded to the quinine treatment until his son informed him that he could have a chill at any time by taking quinine, wherefore he had to abandon its use, and forthwith his son began to improve. A gentleman from an adjoining county informs me of his child having this fever, it being the first of the kind ever seen or treated by the attending physician, who gave it quinine freely after a mercurial purge ; but, as the little patient grew worse all the time, the quinine was suspended, and the child began to improve at once upon the method already mentioned—upon which I have treated a great many cases successfully, rarely ever losing one with this fever, and the treatment upon which I was willing to risk my own life.

From August until October, or later, the fevers along the rivers, creeks, and stagnant waters in Texas are usually very malignant, and often suddenly fatal ; especially is this the case when there has been an abundance of rain in the early part of the season, followed by good crops.

It is highly important, therefore, when called to a case of malarial fever, during this season of the year, to administer a thorough antibilious course, similar to the one mentioned in the first part of this paper, and if it should fail to act promptly it should be worked off with castor oil, Epsom salts, or other saline aperient before administering quinine, unless the case urgently requires the quinine sooner.

As nausea and vomiting usually appear after the fever has progressed a few days, too much care cannot be exercised in order to prevent further irritation of the stomach, which should never be oppressed with drugs. The remedies should be given in the most palatable and pleasant form, and not pressed upon the patient when he cannot retain the same. I reached the point of intolerance for my favorite remedies when a clever clergyman, who visted me daily, insisted upon my taking more chlorate of potassium, but I knew better than he did that I could not bear any more drugging, however simple the remedies seemed to a well person.

In the event of extreme nausea and vomiting of the dark substance that is usual in this fever—which resembles yellow fever in so many respects, and should, therefore, be classed in our nosology as *Icteroid Fever*—the following plan of treatment has never yet failed to give prompt and immediate relief: Into a No. 1 capsule put an ordinary dose of oxalate cerium, add thereto five drops pure creasote, a moderate dose of morphine, and fill the capsule with subnitrate of bismuth. This may be washed down with a swallow of pure wine, or a little brandy diluted and sweetened to the taste, or a swallow of ice-water, though it is more likely to be retained with the stimulant, as suggested. It may be repeated in two, three, or four hours, if necessary, though one or two doses will almost always allay the nausea and retching.

It must not be taken for granted that the patient will always recover after the urine clears up. I have seen one or two cases die from exhaustion after this usually favorable change had taken place. The physician or an experienced, intelligent nurse, should be by the patient's bedside day and night until the crisis is passed, on the lookout for the first warning of outspoken collapse—the cold, heavy, clammy sweat, the small, feeble pulse, almost gone, with cold extremities, increasing stupor and the other plain indications of speedy dissolution. Here, brandy or milk and brandy, if it can be retained, should be given boldly and frequently until the danger is overcome. I would not hesitate to give two or three drops of nitro-glycerine in such an emergency, if the brandy failed.

I cannot close this paper without again condemning, in the most earnest measure, the application of blisters in this fever, which will add only to the suffering of the patient, and the irritation produced thereby was such a shock to the nervous system, in one case, while the blister was being dressed, the frail patient sank into wild deliriums, followed by deep coma and death. * * *

The more I see of this disease the more I am impressed with the importance of pushing the anti-bilious remedies earnestly, in the beginning of the fever, until copious bilious actions follow.

Convalescence is slow and tedious, for the system is very thoroughly depleted after an attack of this fever. Care should be exercised in diet and recreation—never eating anything indigestible, and never taking exercise to fatigue. Some suitable tonic should be taken daily, and, if agreeable, lager beer, wine or brandy, until the health is fully restored.—*Nashville Medical Journal.*

